# Supplementary material for: Common Genetic Variation in MC4R Does Not Affect Atherosclerotic Plaque Phenotypes and Cardiovascular Disease Outcomes
Source: J Clin Med. 2021 Mar 1;10(5):932. doi: 10.3390/jcm10050932 (PMC7957774; doi:10.3390/jcm10050932)
Supplement: Supplementary file 1 [file jcm-10-00932-s001.pdf]

Supplementary Materials

Expanded Methods

General population: Copenhagen City Heart Study and Copenhagen General Population Study

The Copenhagen City Heart Study (CCHS) is a study of the general population that was initiated in 1976–1978 with follow-up examinations in 1981–1983, 1991–1994, and 2001–2003. Participants were selected with the use of the Danish Civil Registration System to reflect the adult Danish population 20 to 100 years of age or older. Data were obtained from a questionnaire, a physical examination, and from blood samples. During follow-up (which ended in May 2011), 2817 participants had incident ischemic vascular disease, of whom 2198 had ischemic heart disease. The Copenhagen General Population Study (CGPS) is a study of the general population that was initiated in 2003 with ongoing enrolment. Participants were recruited and examined exactly as in the CCHS.

Information on diagnoses of ischemic heart disease (International Classification of Diseases, 8th revision [ICD-8], codes 410 through 414; 10th revision [ICD-10], codes I20 through I25) and ischemic cerebrovascular disease (ICD-8, codes 431 through 438; ICD-10, codes I60 through I69 and G45) was collected and verified through a review of all hospital admissions and diagnoses entered in the Danish National Patient Registry, all causes of death entered in the National Danish Causes of Death Registry, and medical records from hospitals and general practitioners. The Danish National Patient Registry has information on all patient contacts with all clinical hospital departments and outpatient clinics in Denmark, including emergency wards (from 1994). The National Danish Causes of Death Registry contains data on the causes of all deaths in Denmark, as reported by hospitals and general practitioners. Ischemic heart disease was fatal or non-fatal myocardial infarction or characteristic symptoms of angina pectoris, including revascularization procedures (ICD8: 410-414; ICD10: I20-I25). A diagnosis of myocardial infarction followed the changing definitions over time and required a typical rise and fall of biochemical markers (troponin or CK-MB), with later changes as indicated. Cases with ischemic cerebrovascular disease and ischemic stroke (ICD8: 431-438; ICD10: I60-I69, G45) were collected likewise.

General population: UK Biobank

The UK Biobank is a study of the general population of the United Kingdom. Baseline assessments took place between 2006 and 2010 in 22 different assessment centers across the country.[28] The project was completed under project number 56340. Information on diagnoses was collected through linkage with the NHS hospital admissions database based on ICD-10 coding (CAD, I21, I22, I24, I25; MI, I21; Stroke, I63 [ischemic stroke only]). More information on the genotyping processes can be found online.[29]

Population at risk for cardiovascular disease: PROSPER

Between December 1997 and May 1999, subjects were screened and enrolled in Scotland (Glasgow), Ireland (Cork), and the Netherlands (Leiden) for the PROspective Study of Pravastatin in the Elderly at Risk (PROSPER). Men and women aged 70-82 years were recruited if they had pre-existing vascular disease or increased risk of such disease because of smoking, hypertension, or diabetes. A total number of 5,804 subjects were randomly assigned to pravastatin or placebo. A large number of prospective tests were performed including Biobank tests and cognitive function measurements. The primary endpoint in the study was the combined endpoint of death from coronary heart disease (CHD), non-fatal myocardial infarct (MI), and occurrence of clinical stroke, either fatal or non-fatal. When death occurred following a non-fatal stroke within a period of 28 days, it was regarded as a fatal stroke. All endpoints were adjudicated by the study endpoint committee. A detailed description of the study has been published elsewhere.[19,30]

A whole genome wide screening has been performed in the sequential PHASE project.[21] Of 5,763 subjects DNA was available for genotyping. Genotyping was performed with the Illumina 660K beadchip, after quality control (call rate <95%) 5,244 subjects and 557,192 SNPs were left for analysis. These SNPs were imputed to 2.5 million SNPs based on the HAPMAP built 36 with MACH imputation

software. Specific SNPs were extracted from the database with Plink software (http://zzz.bwh.harvard.edu/plink/).

Atherosclerotic plaque characteristics: Athero-Express study

Details on genotyping, quality control and imputation have been published previously.[31] In brief, DNA was extracted from whole blood, or alternatively from plaque samples, following standardized in-house validated protocols. Genotyping was done using commercially available genotyping chips. The first batch (Athero-Express Genomics Study 1, AEGS1) was genotyped using Affymetrix Genome-Wide Human SNP Array 5.0, the second batch (Athero-Express Genomics Study 2, AEGS2) was genotyped using the Affymetrix Axiom® GW CEU 1 Array (Affymetrix, Santa Clara, CA, USA). We adhered to community standard quality control and assurance (QCA) procedures to clean the genotype data obtained in AEGS1 (N = 571) and AEGS2 (N = 868).[32] The 998 phased haplotypes from the Genome of the Netherlands Project release 4 (GoNL4) encompassing 19,763,454 SNPs was used as the reference panel for imputation.[33]

Histological sections were stained with haematoxylin and eosin for a general overview including calcifications and intraplaque hemorrhages, picrosirius red for collagen content, α-actin for smooth muscle cells, CD68 for macrophages, CD66b for neutrophils, mast cell tryptase for mast cells, and CD34 for microvessel density. Presence of collagen, and calcification were scored semi-quantitatively as no, minor, moderate or heavy staining in different locations in the plaque, and grouped for analysis in no/minor vs. moderate/heavy; intraplaque hemorrhage was scored as absent (no) vs. present (yes). Fat content was scored as no or less than 10%, and more than 10%. Macrophage and smooth muscle cell content were reported as percentage positive staining per plaque area. Microvessel density in the plaque was quantified in three hotspots and expressed as an average number of vessels per hotspot. In a subsample of the total population, neutrophil and mast cell content was quantified as total number of positive cells in the plaque.

Supplementary table S1. Characteristics of 357,426 British participants of in the UK Biobank, stratified by MC4R rs17782313-C carriage.

	**Non-carriers**	**Heterozygotes**	**Homozygotes**
Number of participants, N (%)	210,184 (59%)	127,658 (36%)	19,584 (6%)
Number of women, N (%)	114,871 (55%)	69,492 (54%)	10,772 (55%)
Age (years)	57 (8)	57 (8)	57 (8)
Body mass index (kg/m2)	27.2 (4.6)	27.5 (4.8)	27.8 (4.9)
Hypertension, N (%)	55,388 (26%)	34,202 (27%)	5,381 (27%)

Values are mean and SD or number of subjects (N) and percentage.

Supplementary table S2. Characteristics of 5241 participants of in the PROspective Study of Pravastatin in the Elderly at Risk (PROSPER), stratified by MC4R rs17782313-C carriage.

	**Non-carriers**	**Heterozygotes**	**Homozygotes**
Number of participants, N (%)	3,152 (60%)	1,814 (35%)	275 (5%)
Number of women, N (%)	1,674 (52%)	934 (52%)	139 (51%)
Age (years)	75 (72-78)	75 (72-78)	75 (72-78)
Body mass index (kg/m2)	26 (24-29)	27 (24-29)	27 (25-30)
Hypertension, N (%)	1,948 (62%)	1,135 (63%)	173 (63%)

Values are median and interquartile range or number of subjects (N) and percentage.

Supplementary table S3. Characteristics of 1439 participants of in the Athero-Express study, stratified by MC4R rs17782313-C carriage.

	Non-carriers	Heterozygotes	Homozygotes
Number of participants, N (%)	798	556	85
Number of women, N (%)	256 (32.1%)	180 (32.4%)	26 (20.6%)
Age (years)	69 (62-76)	69 (63-76)	71 (63-76)
Body mass index (kg/m2)	26 (24-28)	26 (24-28)	26 (24-28)
Hypertension, N (%)	681 (85%)	478 (86%)	72 (85%)

Values are median and interquartile range or number of subjects (N) and percentage.

Supplementary table S4. Effects of *MC4R* rs17782313-C allele carriage on incident cardiovascular disease outcomes, i.e. ischaemic vascular disease (IVD; 18,404 cases) and myocardial infarction (MI; 5,721 cases), in the Copenhagen City Heart Study and the Copenhagen General Population Study (N=106,018 participants; 58,381 women and 47,637 men). Results from three Cox proportional hazards regression models are shown.

Effect allele	Outcome	Covariate added to the standard model[1]	Stratum of sex	Hazard ratio	95% CI
C	IVD	-	Total	1.00	1.03, 0.98
C	IVD	-	Men	1.01	1.04, 0.98
C	IVD	-	Women	0.99	1.03, 0.96
C	IVD	BMI at baseline	Total	1.00	1.02, 0.97
C	IVD	BMI at baseline	Men	1.01	1.04, 0.97
C	IVD	BMI at baseline	Women	0.99	1.02, 0.95
C	MI	-	Total	1.02	1.07, 0.98
C	MI	-	Men	1.04	1.10, 0.99
C	MI	-	Women	0.98	1.05, 0.91
C	MI	BMI at baseline	Total	1.01	1.06, 0.97
C	MI	BMI at baseline	Men	1.04	1.10, 0.98
C	MI	BMI at baseline	Women	0.97	1.04, 0.90

[1] Standard model was adjusted for age and sex (for the total population). BMI, body mass index; SBP, systolic blood pressure.

Supplementary table S5. Effects of MC4R rs17782313-C allele carriage on incident cardiovascular disease outcomes, i.e. coronary artery disease (CAD; 7,560 cases), stroke (1,625 cases) and myocardial infarction (MI; 2481 cases), in the UK Biobank (N=357,426). Results from three Cox proportional hazards regression models are shown.

	**Outcome**	**Covariate added to the standard model[1]**	**Stratum of sex**	**Number of participants**	**Hazard ratio**	**95% CI**
C	CAD	-	Total	347,088	1.03	0.99, 1.07
C	CAD	-	Men	154,637	1.03	0.98, 1.08
C	CAD	-	Women	192,451	1.03	0.96, 1.10
C	CAD	BMI at baseline	Total	347,088	1.02	0.98, 1.06
C	CAD	BMI at baseline	Men	154,637	1.02	0.97, 1.06
C	CAD	BMI at baseline	Women	192,451	1.02	0.95, 1.10
C	Stroke	-	Total	356,717	0.93	0.85, 1.01
C	Stroke	-	Men	161,793	0.95	0.86, 1.05
C	Stroke	-	Women	194,924	0.88	0.77, 1.02
C	Stroke	BMI at baseline	Total	356,717	0.92	0.84, 1.00 *
C	Stroke	BMI at baseline	Men	161,793	0.95	0.85, 1.05
C	Stroke	BMI at baseline	Women	194,924	0.86	0.74, 1.00 *
C	MI	-	Total	354,354	1.01	0.94, 1.08
C	MI	-	Men	159,753	1.00	0.93, 1.08
C	MI	-	Women	194,601	1.03	0.90, 1.17
C	MI	BMI at baseline	Total	354,354	1.01	0.94, 1.07
C	MI	BMI at baseline	Men	159,753	1.00	0.92, 1.08
C	MI	BMI at baseline	Women	194,601	1.03	0.90, 1.17

[1] Standard model was adjusted for age and sex (for the total population). * Statistically significant with P=0.04. BMI, body mass index.

Supplementary table S6. Effects of *MC4R* rs17782313-C allele carriage on incident cardiovascular disease outcomes, i.e. coronary artery disease (881 cases), myocardial infarction (648 cases) and stroke (266 cases), in the PROspective Study of Pravastatin in the Elderly at Risk (PROSPER) (N=5,241 participants; 2,720 women and 2521 men).

Effect allele	Outcome	Covariate added to the standard model a	Stratum of sex	Hazard ratio	95% CI
C	CAD	-	Total	1.05	0.94, 1.18
C	CAD	-	Men	1.07	0.92, 1.25
C	CAD	-	Women	1.02	0.85, 1.22
C	CAD	BMI at baseline	Total	1.05	0.93, 1.18
C	CAD	BMI at baseline	Men	1.07	0.92, 1.25
C	CAD	BMI at baseline	Women	1.02	0.85, 1.22
C	Stroke	-	Total	1.12	0.91, 1.38
C	Stroke	-	Men	1.13	0.84, 1.51
C	Stroke	-	Women	1.11	0.83, 1.50
C	Stroke	BMI at baseline	Total	1.13	0.91, 1.39
C	Stroke	BMI at baseline	Men	1.13	0.84, 1.51
C	Stroke	BMI at baseline	Women	1.12	0.83, 1.51
C	MI	-	Total	1.03	0.90, 1.18
C	MI	-	Men	1.07	0.90, 1.27
C	MI	-	Women	0.97	0.78, 1.21
C	MI	BMI at baseline	Total	1.02	0.89, 1.17
C	MI	BMI at baseline	Men	1.07	0.89, 1.26
C	MI	BMI at baseline	Women	0.96	0.77, 1.20

[1] Standard model was adjusted for age, sex (for the total population) and country of recruitment. BMI, body mass index.

Supplementary table S7. Effects of *MC4R* rs17782313-C allele carriage on atherosclerotic plaque characteristics, in the Athero-Express study (N=1439; 462 women and 977 men).

Effect	Outcome	Covariate added to the standard model[1]	Stratum of sex	N	Beta (SE)	P-value
C	Macrophages	-	Total	1,402	-0.02 (0.02)	0.208
C	Macrophages	-	Men	952	-0.03 (0.02)	0.119
C	Macrophages	-	Women	450	0.00 (0.03)	0.959
C	Macrophages	BMI	Total	1,306	-0.02 (0.02)	0.178
C	Macrophages	BMI	Men	896	-0.03 (0.02)	0.114
C	Macrophages	BMI	Women	410	0.00 (0.03)	0.942
C	Neutrophils	-	Total	253	-0.06 (0.14)	0.676
C	Neutrophils	-	Men	175	-0.08 (0.17)	0.656
C	Neutrophils	-	Women	78	0.02 (0.25)	0.937
C	Neutrophils	BMI	Total	249	-0.04 (0.14)	0.800

C	Neutrophils	BMI	Men	172	-0.06 (0.17)	0.725
C	Neutrophils	BMI	Women	77	0.03 (0.27)	0.923
C	Mast cells	-	Total	210	-0.00 (0.08)	0.983
C	Mast cells	-	Men	149	-0.02 (0.09)	0.857
C	Mast cells	-	Women	61	0.00 (0.16)	0.997
C	Mast cells	BMI	Total	200	0.00 (0.08)	0.993
C	Mast cells	BMI	Men	141	-0.01 (0.09)	0.954
C	Mast cells	BMI	Women	59	-0.03 (0.17)	0.865
C	Smooth muscle cells	-	Total	1,397	0.00 (0.01)	0.994
C	Smooth muscle cells	-	Men	949	0.00 (0.02)	0.880
C	Smooth muscle cells	-	Women	448	0.00 (0.02)	0.889
C	Smooth muscle cells	BMI	Total	1,301	-0.01 (0.01)	0.717
C	Smooth muscle cells	BMI	Men	893	0.00 (0.02)	0.895
C	Smooth muscle cells	BMI	Women	408	-0.01 (0.03)	0.712
C	Collagen content	-	Total	1,413	-0.09 (0.11)	0.403
C	Collagen content	-	Men	961	-0.08 (0.13)	0.530
C	Collagen content	-	Women	452	-0.11 (0.20)	0.581
C	Collagen content	BMI	Total	1,316	-0.10 (0.11)	0.400
C	Collagen content	BMI	Men	904	-0.07 (0.14)	0.589
C	Collagen content	BMI	Women	412	-0.13 (0.20)	0.539
C	Intraplaque haemorrhage	-	Total	1,413	0.09 (0.09)	0.330
C	Intraplaque haemorrhage	-	Men	960	0.13 (0.12)	0.280
C	Intraplaque haemorrhage	-	Women	453	0.06 (0.16)	0.703
C	Intraplaque haemorrhage	BMI	Total	1,316	0.12 (0.10)	0.220
C	Intraplaque haemorrhage	BMI	Men	903	0.16 (0.12)	0.179

C	Intraplaque haemorrhage	BMI	Women	413	0.07 (0.17)	0.666
C	Vessel density	-	Total	1,304	-0.01 (0.00)	0.075
C	Vessel density	-	Men	887	-0.01 (0.01)	0.199
C	Vessel density	-	Women	417	-0.01 (0.01)	0.225
C	Vessel density	BMI	Total	1,213	-0.01 (0.00)	0.113
C	Vessel density	BMI	Men	834	-0.01 (0.01)	0.282
C	Vessel density	BMI	Women	379	-0.01 (0.01)	0.244
C	More than 40% atheroma fat content	-	Total	1,414	0.11 (0.10)	0.262
C	More than 40% atheroma fat content	-	Men	961	0.04 (0.12)	0.723
C	More than 40% atheroma fat content	-	Women	453	0.32 (0.21)	0.124
C	More than 40% atheroma fat content	BMI	Total	1,317	0.13 (0.11)	0.236
C	More than 40% atheroma fat content	BMI	Men	904	0.04 (0.12)	0.749
C	More than 40% atheroma fat content	BMI	Women	413	0.40 (0.22)	0.074
C	More than 10% atheroma fat content	-	Total	1,414	-0.06 (0.10)	0.532
C	More than 10% atheroma fat content	-	Men	961	-0.12 (0.13)	0.331
C	More than 10% atheroma fat content	-	Women	453	0.03 (0.17)	0.847
C	More than 10% atheroma fat content	BMI	Total	1,317	-0.07 (0.10)	0.529
C	More than 10% atheroma fat content	BMI	Men	904	-0.13 (0.13)	0.332
C	More than 10% atheroma fat content	BMI	Women	413	0.03 (0.17)	0.853

C	Calcification	-	Total	1,412	-0.13 (0.09)	0.151
C	Calcification	-	Men	959	-0.18 (0.11)	0.094
C	Calcification	-	Women	453	-0.03 (0.16)	0.858
C	Calcification	BMI	Total	1,315	-0.09 (0.09)	0.318
C	Calcification	BMI	Men	902	-0.15 (0.11)	0.178
C	Calcification	BMI	Women	413	0.03 (0.17)	0.871

[1] Standard model was adjusted for age, sex (for the total population), cohort, year of surgery and two principle components. BMI, body mass index.
